# A second species of the North American mayfly genus Amercaenis Provonsha and McCafferty (Ephemeroptera:Caenidae)

**DOI:** 10.1673/1536-2442(2006)6[1:ASSOTN]2.0.CO;2

**Published:** 2006-06-26

**Authors:** A. V. Provonsha, W. P. McCafferty

**Affiliations:** Department of Entomology, Purdue Unviversity, West Lafayette, IN 47905

**Keywords:** *Amercaenis cusabo*, new species, *Amercaenis ridens*, Southeastern United States

## Abstract

A new species of the psammophilous and brushlegged North American genus Amercaenis Provonsha and McCafferty, Amercaenis cusabo Provonsha and McCafferty, new species, is described from larvae taken from the Black River (North Carolina), the Pee Dee River (North Carolina), and the Savannah River (Georgia and South Carolina). The new species differs from the only other known species of the genus, A. ridens (McDunnough), for example, in having segments 2 and 3 of the labial palps subequal in length, and having the transverse row of setae on the forefemora consisting of long spatulate setae. Adults provisionally assigned to the new species are associated with the larvae morphologically and in locale do not fit any other adults of North American Caenidae, and differ from the congener A. ridens by dark dorsal coloration. The two species of Americaenis are biogeoraphically disjunct.

## Introduction

The cosmopolitan mayfly family Caenidae, the small squaregill mayflies, was last reviewed by McCafferty and Wang (2000). At that time, the family was divided into the three subfamilies Brachycercinae, Caeninae, and Madecocercinae. The subfamily Caeninae is by far the most diverse and widespread and most recently has included 12 valid genera (Suter 1999; McCafferty and Wang 2000; Sun and McCafferty 2001, 2004). Among these 12 genera, three have been known as “brushlegged” caenid mayflies (Provonsha and McCafferty 1995) because of the extremely long and profuse hairlike setae present on the forelegs of the larvae. The brushlegged caenid genera include ClypeocaenisSoldán (1978) from Africa and the Orient, AmercaenisProvonsha and McCafferty (1985) from North America, and Barnardara McCafferty and Provonsha (Provonsha and McCafferty 1995) from eastern and southern Africa.

Several characters separate Amercaenis from the other brushlegged genera. For example, in Amercaenis larvae, long foreleg setae are present only along the inner margins of the tibia and tarsus, whereas in Clypeocaenis, these setae are arranged in uniform diagonal rows, and in Barnardara, such setae are randomly scattered over the tibia and tarsus. For other distinguishing characteristics among the brushlegged caenines, see Provonsha and McCafferty (1985, 1995).

Amercaenis has been known from only one species, A. ridens (McDunnough), a psammophilous species known only from the central lowlands, presently the U.S. states of Iowa, Kansas, Missouri, Nebraska, and Texas (e.g., McCafferty et al. 2003). In the larval stage, Amercaenis is distinguished from all other caenine mayflies in North America by the presence of long foreleg setae; highly setose labial palps; the presence of short spatulate setae on the surface of the mid- and hindfemora and on the surface of the operculate gills; and the absence of long simple setae along the posterior margins of the operculate gills and abdominal terga 7–8. To date, no individual characters have been found that will consistently separate adults of Amercaenis from all North American species of Caenis (Provonsha, 1990). Adults of Amercaenis species are separable among all Caeninae, however, with the use of a combination of characteristics (see below).

In recent years we have seen material of a new distinctive species of Amercaenis taken from sandy substrate habitats in the Black River and Pee Dee River (North Carolina) and the Savannah River (Georgia and South Carolina). It is our purpose in this paper to describe this morphologically and geographically distinct second-known species of the genus.

## Taxonomy Amercaenis cusabo, new species

### Mature larva

Body length 4.2 mm.

**Figure 1–9.**
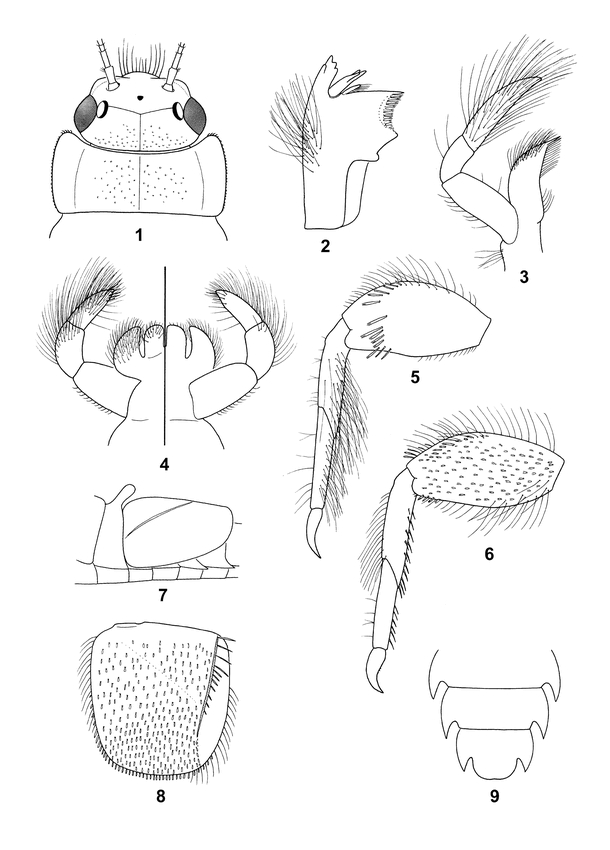
Amercaenis cusabo, larva. 1, Head and pronotum, dorsal. 2, Angulate mandible. 3, Maxilla. 4, Labium. 5, Foreleg, dorsal. 6. Hindleg, dorsal. 7, Base of abdomen, lateral. 8, Operculate gill, dorsal. 9, Abdominal sterna 7–9.

*Head:* Dorsal coloration uniform dark chestnut brown; ventral coloration pale; genal ridge (Fig. 1) slightly developed; clypeus slightly produced anteriorly, rounded, with numerous long hair-like setae (Fig. 1); vertex with small, globulate microtrichiae (Fig. 1). Planate and angulate mandible (Fig. 2) with cluster of long hair-like setae near outer margin. Maxilla (Fig. 3) with three-segmented palp; palp segment 2 less than half length of segment 3; palp segment 3 and crown of galealacinia setose; sparse setae of palp segment 2 and dense setae of palp segment 3 relatively long; sparse setae of palp segment 1 and dense setae of crown shorter. Labium (Fig. 4) with three-segmented palp; palp segments 2 and 3 subequal in length, covered with dense, long, hair-like setae; palp segment 3 also with short, distal, spine-like setae.

*Thorax:* Nota uniform dark chestnut brown; sterna pale. Pronotum (Fig. 1) lateral margins subparallel over most of length; anterior margin concave and juxtaposed with posterior convex margin of the head; anterolateral corners with short, stout bristles. Tibiae light chestnut brown basally, becoming pale distally; all tarsi pale. Fore femur (Fig 5) coloration pale with suffused dark brown band near distal margin; dorsal surface with transverse row of prominent spatulate setae at approximately two-thirds length from base; posterior (outer) margin with long simple setae. Fore tibia and fore tarsus (Fig. 5) with dense, long, hair-like setae anteriorly (inner margin) and subanteriorly; fore claw (Fig 5) long, curved at tip, and adenticulate. Mid- and hind femora suffused with chestnut brown, darkest distally; anterior (inner) and posterior (outer) margins (Fig. 6) with moderately long, hair-like setae; surface covered with small spatulate setae. Mid- and hind tibiae (Fig. 6) with moderately long, hair-like setae along outer margin; mid- and hind tibiae and mid- and hind tarsi (Fig. 6) with short, bristle-like setae along inner margin; mid- and hind tarsi (Fig. 6) with only sparse short setae on outer margin; mid-and hind claws (Fig. 6) with central row of three to five minute denticles on inner margin.

*Abdomen:* Segments with lateral margins with rows of moderately long setae; segments 3–9 with well-developed posterolateral projections (Fig. 9). Terga 1 dark chestnut brown medially, pale laterally; terga 2 with well-developed dorsoposterioly directed and apically rounded median projection (Fig. 7); terga 3–9 pale medially, dark chestnut brown laterally, with large black-brown spot at base of lateral projection; tergum 10 mostly pale; terga 6 and 7 posterior margins with moderately long, posteriorly directed, spatulate, bifurcate setae. Sterna mostly pale, chestnut brown laterally and along anterior margin; sternum 9 broadly rounded, or slightly emarginated along posterior margin (Fig. 9). Gill 1 short, subequal in length to midlength of tergum 2. Operculate gill (Figs. 7, 8) dorsal coloration uniform dark chestnut brown; medial and outer margins with rows of hair-like setae as in Fig. 8; posterior margin with short, spatulate setae (Fig. 8); dorsal surface covered with short spatulate setae; median fork of Y-ridge raised and slightly keel shaped and with row of medially directed setae as in Fig. 8; outer fork of Y-ridge barely discernible. Caudal filaments with long lateral setae at juncture of each segment; such setae longer than adjacent segment.

### Male adult (in alcohol)

Body length 2.0–2.5 mm.

**Figure 10–12.**
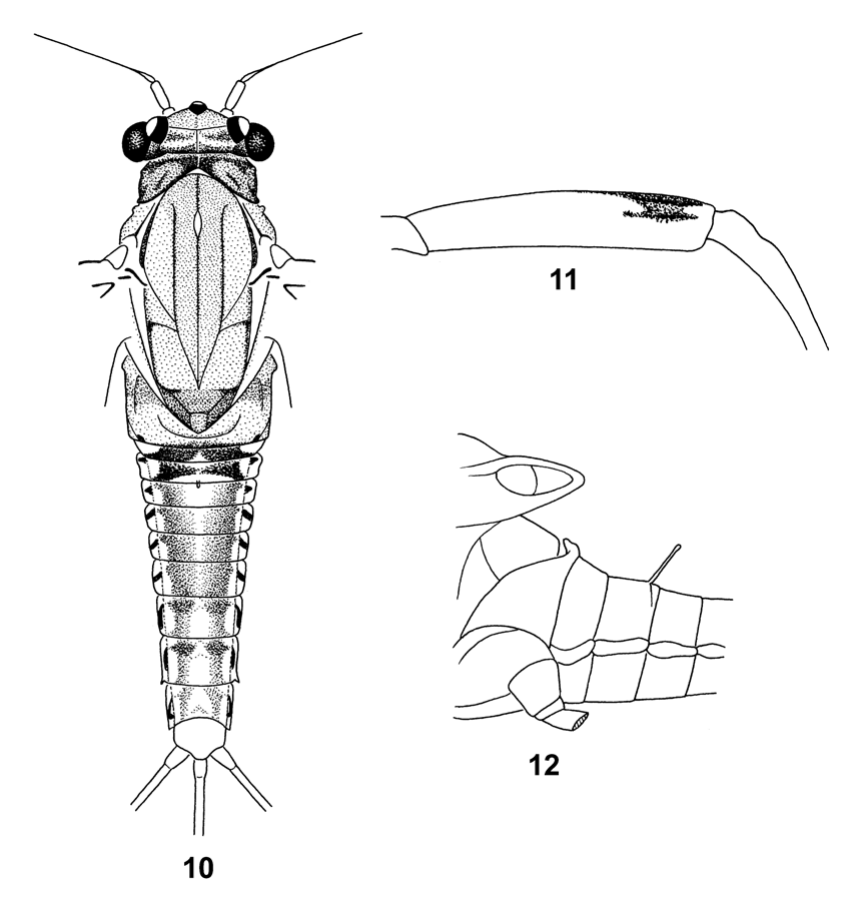
Amercaenis cusabo, male adult. 10, Body, dorsal. 11, Hind femur. 12, Base of abdomen, lateral.

*Head:* Vertex (Fig. 10) shaded with dark brown, and with medium brown band between lateral ocelli; anterior and posterior margins of head capsule edged with dark brown. Antennae pale.

*Thorax:* Pronotum marked with dark brown as in Fig. 10. Mesonotum (Fig. 10) tan, with median, anterior parapsidal, and prescutal sutures heavily marked with dark brown; scutellum dark brown; mesosternum pale yellow. Wings with SC and R1 veins dark brown; all other wing veins pale. Forelegs stained with medium brown and dark brown distally on all segments. Mid- and hind femora (Fig. 11) with distinct marking subdistally as in Fig. 11.

*Abdomen*: All terga (Fig. 10) with pale lateral margins; terga 1–2 dark brown; tergum 2 with long, thin, pencil shaped median projection (Fig. 12); terga 3–6 (Fig. 10) stained with light brown over much of cuticle, variously pale medially—moreso anteriorly; terga 7–8 light brown in anterior third, with darker brown, sublateral, broad, longitudinal brown bars as in Fig. 10; tergum 9 somewhat similar to 7 and 8, but with brown at posterior margin between sublateral bars; tergum 10 pale. Laterally extended pleura pale, with various shaped black macula as in Fig. 10. Sterna pale, unmarked. Genital forceps short, flattened, and densely covered with microspines; similar to A. ridens and members of the Caenis hilaris group. Caudal filaments pale.

### Female adult (in alcohol)

Body length 2.5 mm.

Projection on abdominal tergum 2 similar to that of male. Coloration and markings as described for male.

### Egg

Chorion with single, type I polar cap (see Provonsha, 1990).

### Material examined

Holotype: larva in alcohol, North Carolina, Bladen Co, Black R, VIII-1993, deposited in the Purdue Entomological Research Collection, West Lafayette, IN (PERC). Paratypes: two larvae, same data and deposition as holotype. Other material examined: One larva, Georgia, Burke Co & South Carolina, Barnwell Co, Savannah R, ANSP Savannah R Proj (SRP), Sta 5, VIII-29-1968, J Richardson, deposited Academy of Natural Sciences, Philadelphia (ANSP); one larva, Georgia, Richmond Co & South Carolina, Aiken Co, Savannah R, ANSP SRP#5, Sta 1, VIII-25-1955, S Roback (ANSP); Three larvae, Georgia, Screven Co & South Carolina, Allendale Co, Savannah R, ANSP SRP#6, Sta 6, VIII-21-1955, S Roback, IX-15-1992, IX-14-1993, E Silldorff (PERC, ANSP); four larvae, Georgia, Burke Co & South Carolina, Barnwell Co, Savannah R, ANSP SRP#1, Sta 3, VI-30-1951, IX-14-1993 (PERC, ANSP); three larvae, North Carolina, Anson & Richmond Cos, Pee Dee R, upstream from entrance to Staples Lake Landing nr dam, VIII-08-2001, R Smith (PERC); 57 male and one female adults, Georgia, Screven Co & South Carolina, Allendale Co, Savannah R at Hwy 301 bridge, IX-28-1994, E Silldorff (PERC, ANSP); one male adult, Georgia, Richmond Co & South Carolina, Aiken Co, Savannah R, R Mile 157, IX-27-1994, E Silldorff (ANSP).

### Etymology

The specific epithet is after the extinct native American people known as the Cusabo tribe that once inhabited part of the South Carolina region.

## Discussion

Amercaenis cusabo and A. ridens are apparently widely disjunct geographically, with the former associated with the southeastern Savannah River drainage system, and the latter associated with the central Missouri and Mississippi drainage systems. Certain other strictly psammophilous species, however, are found in both central and southeastern areas, for example, Spinadis simplex (Walsh) in the family Heptageniidae and Acanthametropus pecatonica (Burks) in the family Acanthametropodidae, although these latter two are not known from the Missouri system.

As larvae, the two species of Americaenis can be easily differentiated from each other morphologically. Segments 2 and 3 of the labial palps of A. cusabo are subequal in length (Fig. 4), whereas segment 3 is over twice as long as segment 2 in A. ridens (Fig. 5, Provonsha and McCafferty 1985). The longer palp segment 3 was attributed to the genus in general by Provonsha and McCafferty (1985) based on only one species and should no longer be considered a diagnostic character for the genus. The transverse row of spatulate setae on the dorsal surface of the fore femora are short in A. ridens (Fig. 6, Provonsha and McCafferty 1985) and much longer in A. cusabo (Fig. 5). The marginal simple setae on all femora of A. cusabo (Figs. 5, 6) are much longer and denser than on A. ridens (Figs. 6, 7, Provonsha and McCafferty 1985). The frons of A. cusabo is more developed (Fig. 1) than that of A. ridens (Fig. 1, Provonsha and McCafferty 1985). Also in general, the body of A. cusabo appears somewhat broader and more depressed than that of A. ridens.

The adults we assign to A. cusabo were not based on reared insects, but were associated with larvae collected from the same locales and having the predictable thin medial projection of abdominal tergum 2 as found in the larvae. Although our assignment of adults to A. cusabo is provisional, we are confident that the association is correct. The adults do not fit any other adults of North American Caenidae, a stage and group that are very well known.

On the bases of individual characters, adults of Amercaenis cannot consistently be distinguished from all North American species of Caenis (Provonsha 1990). However, the dorsal projection on abdominal segment 2 separate Amercaenis from all but C. youngi and C. macafferti. Amercaenis cusaba adults are differentiated from C. youngi by their much smaller size; short, flattened, blade-like forcepts in the male; and single capped eggs in the female. Amercaenis cusabo can easily be distinguished from C. macafferti by its significantly different color pattern. Adults of A. cusabo are distinguishable from the adults of A. ridens by the extensive dark brown color pattern of the abdominal terga in the former.
